# Incidence of diabetes mellitus and associated risk factors in the adult population of the Basque country, Spain

**DOI:** 10.1038/s41598-021-82548-y

**Published:** 2021-02-04

**Authors:** Inés Urrutia, Alicia Martín-Nieto, Rosa Martínez, J Oriol Casanovas-Marsal, Anibal Aguayo, Juan del Olmo, Eunate Arana, Elsa Fernandez-Rubio, Luis Castaño, Sonia Gaztambide, The Diabetes Epidemiology Group, Alejandro García-Castaño, Sara Gómez-Conde, Sara Larrauri, Idoia Martínez de LaPiscina, Laura Saso, Olaia Velasco

**Affiliations:** 1 Biocruces Bizkaia Health Research Institute, Cruces University Hospital, University of the Basque Country UPV/EHU, Leioa, Bizkaia Spain; 2 CIBERDEM (Spanish Biomedical Research Centre in Diabetes and Associated Metabolic Disorders), CIBERER (Spanish Biomedical Research Centre in Rare Diseases), Instituto de Salud Carlos III, Madrid, Spain; 3 Endocrinology and Nutrition Department, Cruces University Hospital, Osakidetza, Bilbao, Bizkaia Spain; 4 Clinical Chemistry Laboratory, Cruces University Hospital, Osakidetza, Bilbao, Bizkaia Spain

**Keywords:** Endocrinology, Health care, Medical research, Risk factors

## Abstract

The aim of this study was to estimate the incidence of diabetes mellitus in the Basque Country and the risk factors involved in the disease by reassessing an adult population after 7 years of follow-up. In the previous prevalence study, 847 people older than 18 years were randomly selected from all over the Basque Country and were invited to answer a medical questionnaire, followed by a physical examination and an oral glucose tolerance test. In the reassessment, the same variables were collected and the resulting cohort comprised 517 individuals of whom 43 had diabetes at baseline. The cumulative incidence of diabetes was 4.64% in 7 years and the raw incidence rate was 6.56 cases/1000 person-years (95%CI: 4.11–9.93). Among the incident cases, 59% were undiagnosed. The most strongly associated markers by univariate analyses were age > 60 years, dyslipidaemia, prediabetes and insulin resistance. We also found association with hypertension, obesity, family history of diabetes and low education level. Multivariate analysis adjusted for age and sex showed that a set of risk factors assessed together (dyslipidaemia, waist-to-hip-ratio and family history of diabetes) had great predictive value (AUC-ROC = 0.899, 95%CI: 0.846–0.953, *p* = 0.942), which suggests the need for early intervention before the onset of prediabetes.

## Introduction

Diabetes mellitus is a chronic disease with an increasing frequency over the last decade related to the aging population and lifestyle changes that promote obesity^1^. Based on current data from worldwide studies, the International Diabetes Federation (IDF) estimates that the global number of people with diabetes (aged 18–99 years) will increase to 693 million by 2045^1^. This serious pathology has become a priority health problem in the twenty-first century according to the World Health Organization (WHO)^2^. Its growing prevalence is predicted to increase the significant social and economic impact resulting from the disease associated complications^2–6^. Nevertheless, prevention is possible when acting on risk factors and making an early diagnosis and adequate treatment that prevent long-term complications^7–9^.

Studies on the diabetes incidence provide more reliable information on the risk factors involved in the disease progression compared to the prevalence ones. Therefore, IDF advocates that accurate incidence studies should be made^1^. Following this recommendation, we have re-evaluated the current health status of the same cohort than previously analysed in the diabetes prevalence study carried out during 2010–2012 in the Basque Country^10^, to determine the incidence of diabetes in our population and the risk factors that are involved in the progression of the disease.

## Results

The reassessment of the cohort that participated in the previous prevalence study (baseline study) after seven years of follow-up, made the determination of diabetes incidence in the Basque Country possible, as well as the evaluation of the risk factors involved in the progression of the disease. The baseline characteristics of participants and non-participants in the present re-examination are shown in Table 1. Except for the obesity (16.8% vs. 23.0%, *p* = 0.025) and diabetes percentage (8.4% vs. 14.3%, *p* = 0.010) that are lower in the group of participants, the frequency of the rest of the variables were similar in both groups.

**Table 1:** Baseline characteristics of the population under study (2010–2012).

	Non-participants	Participants	*p*-value
Sex (men)	152/330 (46.1%)	226/517 (43.7%)	0.549^a^
Age (years)	52.7 (37.6–66.6)	52.3 (40.4–63.5)	0.843^b^
Obesity	77/330 (23.3%)	87/517 (16.8%)	0.025^a^
Abdominal obesity (WC)	181/330 (54.8%)	265/516 (51.4%)	0.357^a^
Abdominal obesity (WHR)	81/330 (24.5%)	111/516 (21.5%)	0.345^a^
Hypertension	157/330 (47.6%)	223/517 (43.1%)	0.231^a^
High triglycerides level	48/320 (15.0%)	98/510 (19.2%)	0.145^a^
Low HDL-cholesterol	47/319 (14.7%)	68/507 (13.4%)	0.667^a^
High LDL-cholesterol	85/316 (26.9%)	154/502 (30.7%)	0.281^a^
Family history of diabetes	126/305 (41.3%)	222/493 (45.0%)	0.339^a^
Education level (primary or lower)	115/330 (34.8%)	166/517 (32.1%)	0.453^a^
Regular exercise (at least once a week)	155/330 (47.0%)	265/517 (51.3%)	0.252^a^
Smoker (> 1 cigarette/day)	80/330 (24.2%)	121/517 (23.4%)	0.844^a^
Normoglycaemia	243/322 (75.5%)	411/513 (80.1%)	0.133^a^
Prediabetes	33/322 (10.2%)	59/513 (11.5%)	0.653^a^
Diabetes	46/322 (14.3%)	43/513 (8.4%)	0.010^a^

Comparison between non-participants and participants in the follow-up.

Age is shown as median (IQR: P_25_–P_75_). Rest of variables is shown as n/total (%) where total is the number of non-participants or participants for each variable, as appropriate. Family history of diabetes defined as 1st or 2nd degree relatives with diabetes. Definitions of the rest of cardiovascular risk factors are detailed in methods.

^a^Pearson’s Chi-square test.

^b^Mann-Whitney U-test.

Among the 517 people who agreed to participate in the reassessment, 43 had diabetes at baseline (2010–2012) and were excluded from all the diabetes incidence calculations. Thus, the group at risk was established with 474 people of whom 22 developed diabetes over a period of 7 years. Among the 22 newly diagnosed diabetes cases, 13 were not aware about their disease (unknown diabetes). All these undiagnosed cases underwent a 75 g OGTT: 5 cases were diagnosed with diabetes by fasting blood glucose, 6 cases by glucose at 120 min and 2 cases by both, fasting and 120 min glucose, according to the WHO criteria^11^. These data yield a cumulative incidence of diabetes of 4.64% in 7 years and a raw incidence rate of 6.56 cases/1000 person-year (95%CI 4.11–9.93). The adjusted estimation for age and sex structure of the Spanish population was of 5.37 cases/1000 person-year (95% CI 5.35–5.40).

The incidence rates of diabetes according to the variables tested in the study, as well as the OR (95%CI) for diabetes resulted from the univariate binomial regression logistic analyses are detailed in Table 2. Although the incidence rate was slightly higher in men, the difference between sexes did not reach statistical significance. The strongest association with the diabetes progression was found from 60 years of age onwards, dyslipidaemia, prediabetes at baseline and insulin resistance. A statistically significant increase in the incidence rate of diabetes in individuals with hypertension, family history of diabetes, low education level and obesity was also found. Other analysed variables, such as physical activity or smoking, failed to show any association with diabetes development.

**Table 2:** Diabetes incidence rates and OR (95% CI) for diabetes according to possible risk factors.

	People at-risk (n)	New DM cases (n)	Person-year (n)	Incidence rate/1000 person-years (95%CI)	OR (95% CI)	*p-*value
Whole cohort	474	22	3356	6.56 (4.11–9.93)	_	_
**Sex**						
Women	282	12	1997	6.01 (3.11–10.50)	1	
Men	192	10	1359	7.36 (3.53–13.53)	1.25 (0.53–2.89)	0.611
**Age (years)**						
18–45	114	1	807	1.24 (0.03–6.90)	1	
46–60	150	4	1062	3.77 (1.03–9.64)	2.32 (0.42–23.41)	0.348
61–75	150	12	1062	11.30 (5.84–19.74)	6.83 (1.61–63.36)	0.006
≥ 76	60	5	425	11.77 (3.82–27.47)	7.5 (1.45–74.4)	0.015
**BMI (Kg/m**^**2**^**)**						
< 25	164	4	1161	3.44 (0.94–8.82)	1	
25–30	207	7	1466	4.78 (1.92–9.84)	1.33 (0.42–4.78)	0.631
≥ 30	100	10	708	14.12 (6.77–25.98)	4.14 (1.40–14.33)	0.010
**Abdominal obesity (WC)**						
No	155	4	1097	3.64 (0.99–9.33)	1	
Yes	232	16	1643	9.74 (5.57–15.82)	2.57 (0.96–8.44)	0.062
**Abdominal obesity (WHR)**						
No	270	6	1912	3.14 (1.15–6.83)	1	
Yes	117	14	828	16.90 (9.24–28.36)	5.7 (2.28–15.84)	< 0.001
**Hypertension**						
No	217	5	1536	3.25 (1.06–7.59)	1	
Yes	185	16	1310	12.22 (6.98–19.84)	3.76 (1.49–11.10)	0.005
**High triglycerides level**						
No	330	10	2336	4.28 (2.05–7.87)	1	
Yes	56	10	396	25.22 (12.09–46.38)	6.89 (2.75–17.32)	< 0.001
**Low HDL-cholesterol**						
No	273	3	1933	1.55 (0.32–4.54)	1	
Yes	126	18	892	20.18 (11.96–31.89)	13.18 (4.60–50.70)	< 0.001
**High LDL-cholesterol**						
No	252	5	1784	2.80 (0.91–6.54)	1	
Yes	147	16	1041	15.37 (8.79–24.97)	5.65 (2.22–16.69)	< 0.001
**Glucose at baseline**						
NFG and/or NGT	415	10	2938	3.40 (1.63–6.26)	1	
Isolated IGT	37	6	262	22.90 (8.41–49.85)	7.97 (2.67–22.29)	< 0.001
Isolated IFG	19	4	135	29.74 (8.10–76.13)	11.21 (3.04–36.41)	< 0.001
IFG and IGT	3	2	21	94.16 (11.4–340.15)	64.37 (7.89–750.91)	< 0.001
**Insulin resistance (HOMA-IR index)**						
No	279	5	1975	2.53 (0.82–5.91)	1	
Yes	106	15	750	19.99 (11.19–32.97)	8.45 (3.28–25.24)	< 0.001
**Family history of diabetes**						
No	192	5	1359	3.68 (1.19–8.58)	1	
Yes	191	15	1352	11.09 (6.21–18.30)	2.99 (1.17–8.89)	0.021
**Education level (primary or lower)**						
No	273	8	1933	4.14 (1.79–8.16)	1	
Yes	114	12	807	14.87 (7.68–25.97)	3.81 (1.56–9.72)	0.003
**Regular exercise (at least once a week)**						
Yes	217	9	1536	5.86 (2.68–11.12)	1	
No	170	11	1204	9.14 (4.56–16.35)	1.58 (0.65–3.92)	0.307
**Smoker (> 1cigarette/day)**						
Non-smoker	159	8	1126	7.11 (3.07–14.00)	1	
Ex-smoker	163	10	1154	8.67 (4.16–15.94)	1.22 (0.48–3.18)	0.676
Smoker	65	2	460	4.35 (0.53–15.70)	0.70 (0.13–2.63)	0.620

People at risk were those without diabetes in the prevalence study (2010–2012). Family history of diabetes defined as 1st or 2nd degree relatives with diabetes. Definitions of the rest of cardiovascular risk factors are detailed in methods. OR (95%CI) were calculated for each variable by univariate binomial logistic regression analyses.

The results of the multivariate binomial logistic regression analyses adjusted for sex and age are shown in Table 3. Two different models were proposed to predict diabetes and were based on the inclusion or not of variables related to the prediabetes symptoms at baseline. In model A, waist-to-hip ratio and family history of diabetes were independently associated with diabetes, together with low HDL-cholesterol and prediabetes at baseline. These two last variables were the strongest diabetes progression predictors in this first proposal. Model B was built with the same variables than the first one except for those with prediabetes and/or insulin resistance. In this second proposal, low HDL-cholesterol was the strongest predictor and together with high triglycerides level, waist-to-hip ratio and family history of diabetes, was associated with diabetes. The area under the receiver-operating-characteristic curve for diabetes was 0.927 (95% CI: 0.891–0.963) in model A and 0.899 (95% CI: 0.848–0.953) in model B. Both models had adequate goodness-of-fit (*p* = 0.613 and *p* = 0.942, respectively).

**Table 3:** Multivariate binomial logistic regression models to assess the contribution of different variables in diabetes prediction.

Model A	OR (95% CI)	*p-*value
Sex (Men)	2.02 (0.65–6.43)	0.224
Age	1.01 (0.97–1.06)	0.660
Abdominal obesity (WHR)	3.93 (1.18–14.20)	0.025
Low HDL-cholesterol	6.66 (2.10–27.18)	< 0.001
Family history of diabetes	3.61 (1.23–12.47)	0.019
Pre-diabetes	7.04 (2.52–20.59)	< 0.001

Model B	OR (95% CI)	*p-*value
Sex (Men)	2.68 (0.91–8.26	0.074
Age	1.03 (0.99–1.07)	0.198
Abdominal obesity (WHR)	3.67 (1.19–12.27)	0.024
High triglycerides level	3.55 (1.29–9.77)	0.014
Low HDL cholesterol	7.08 (2.25–28.82)	< 0.001
Family history of diabetes	3.01 (1.09–9.48)	0.033

The starting model A included all variables with *p* < 0.2 in the univariate analysis. In model B, the same variables were included, except for the pre-diabetes status at baseline and the HOMA-IR index. The two models were built manually. At each step of the modelling process, we repeatedly removed the variable with the highest *p*-value and we tested the model again until all remaining variables were significant (*p* < 0.05). Age and sex, regardless of their statistical significance, were kept in the final model. Family history of diabetes defined as 1st or 2nd degree relatives with diabetes. Definitions of the rest of cardiovascular risk factors are detailed in methods.

Apart from the 22 new cases of diabetes, there were 30 new cases of prediabetes, meaning that overall, 52 people had some glucose metabolism disorder after the follow-up period. In contrast, among the 102 cases with any glucose metabolism disturbance in the previous prevalence study, 36 showed an improvement after the reassessment: 31 people had normoglycaemia and five cases with diabetes had improved to prediabetes.

## Discussion

The current study, based on a representative sample of the Basque Country, estimates an incidence rate of diabetes of 6.56 cases/1000 person-year. The single previous incidence study on diabetes in general population of the Basque Country was carried out 20 years ago^12^, when a cohort of 594 people without diabetes at baseline (> 30 years) was reassessed after a follow-up of 10 years. The estimated raw incidence rate was 7.0 cases/1000 person-year, a little higher than the current estimation. Considering that in the present analysis the only difference between participants and non-participants is the lower percentage of obesity among participants (23% vs. 17%, *p* = 0.026) and, as obesity is a major risk factor in the development of diabetes, we could think that the current incidence may be underestimated and then, assume that the incidence of diabetes in our area has remained stable for 20 years.

It should be noted the difficulty to compare the incidence estimations from different areas due to the varying procedures among studies. However, the present study focused on a region in northern Spain has followed the same methodology than the recently published nationwide study, which tolerates valid comparisons. Our estimated incidence of diabetes in the Basque Country adjusted for the age and sex structure of the Spanish population is 5.37 cases/1000 person-years (95%CI 5.35–5.40), lower than the recently published data for Spain^13^, 11.6 cases/1000 person-years (95%CI 11.1–12.1). There are few data about the incidence of diabetes in general population of different regions in Spain. Despite small differences in the methodology, we could say that the incidence rate in our cohort, 6.56 cases/1000 person-year, is lower than that reported in a neighboring region of northern Spain^14^, 10.8 cases/1000 person-years, and noticeably lower than the estimate for the southern Spain^15^, 19.1 cases/1000 person-years. This fact confirms a heterogeneous distribution of diabetes in the country^16^ and may be related to the prevalence of obesity which is almost half in the Basque Country^10^ compared to southern Spain^17^, 19% versus 37%, respectively.

Unlike many published studies in which the incidence of diabetes is estimated from questionnaires or by reviewing clinical records^18,19^, in our study, diabetes was diagnosed in general population on basis of an OGTT or with a fasting glucose measurement. This procedure, which was carried out both in the baseline study and in the follow-up, allows a more accurate estimation of the incidence of diabetes, since it also includes cases of unknown diabetes. In this survey, more than half of the new diabetes cases, 13/22, were not aware about their disease. This is a disturbing figure that has not improved since the prevalence study in which the unknown diabetes figure accounted for 41% of the diabetes cases^10^. The IDF warns that the risk of developing cardiovascular events is higher in these people not receiving neither treatment nor preventive measures^20^ thereby, it is of great importance to perform regular diabetes screening in general population to reduce the number of unknown cases.

As expected, several cardiovascular risk factors such as the age from 60 onwards, dyslipidaemia, hypertension, obesity and altered WHR, are associated with diabetes in the univariate analyses. However, male sex which is widely defined as a risk factor for developing diabetes, does not reach statistical significance in our study, probably because of the limited number of new cases detected in our cohort. The strongest association with the progression of the disease is found with the prediabetes status at baseline which is greater in isolated cases of IFG than in isolated cases of IGT, as described in other reports^14,21^. This is not an unexpected result considering that the metabolic abnormalities underlying both disorders are different^22^. Individuals with isolated IFG and isolated IGT show similar impairments in insulin action, but those with isolated IFG have a more pronounced defect in early insulin secretion and, additionally, have clear abnormalities in hepatic glucose regulation. More severe metabolic abnormalities are present in individuals with combined IFG and IGT, being the most strongly associated disorder with the progression of diabetes in our cohort.

All tested variables in the univariate analyses were used to build two multivariate models. The model including variables related to the symptoms of prediabetes at baseline has the highest predictive capacity, but the other has a high predictive value too. This second model supports the importance of abdominal obesity, dyslipidaemia and the genetic component in the etiopathogenesis of the disease and suggests the need for early intervention before the onset of prediabetes to prevent the progression of the pathology.

Specially worth mentioning is that in our cohort, 30% of people with some glucose metabolism disorder at baseline reverted to normoglycaemia after 7 years of follow-up. This figure is similar to that found in other studies^8,23^. This percentage of reversion could be due to the less strict criteria used in epidemiology for the diagnosis of diabetes, which results in false positives. However, it should not be ruled out that weight loss may contribute to improving glucose metabolism, highlighting the potential of non-pharmacological prevention strategies^9,24,25^.

It could be considered a limitation of the study that 33% of people of the original cohort were lost to follow-up. However, the participation of those contacted reached 85% and few differences were found between participants and non-participants, minimizing any possible selection bias. Additionally, only 19% of participants refused the blood analysis and completed exclusively the basic questionnaire for diagnosis. The main strengths of our study are, first, the methodology that has allowed valid comparisons with recently published data at national level, and second, the sampling. Indeed, this is the first study carried out on the general population in different geographical areas of the Basque Country and therefore has a more accurate estimate of the incidence of diabetes in the Basque population, including also undiagnosed cases.

In summary, the incidence rate of diabetes in the Basque Country remains stable over time and lower than those reported from other Spanish regions probably due to the lower percentage of obesity. Furthermore, our results show that the criteria for identifying the population at high risk of developing diabetes should not be based exclusively on the presence of prediabetes, but the predictive value of a set of risk factors assessed together, such as dyslipidaemia, waist-to-hip ratio and family history of diabetes support enough evidence to approach a lifestyle intervention that avoids the progression of the disease.

## Patients and methods

### Population

**Figure 1:**
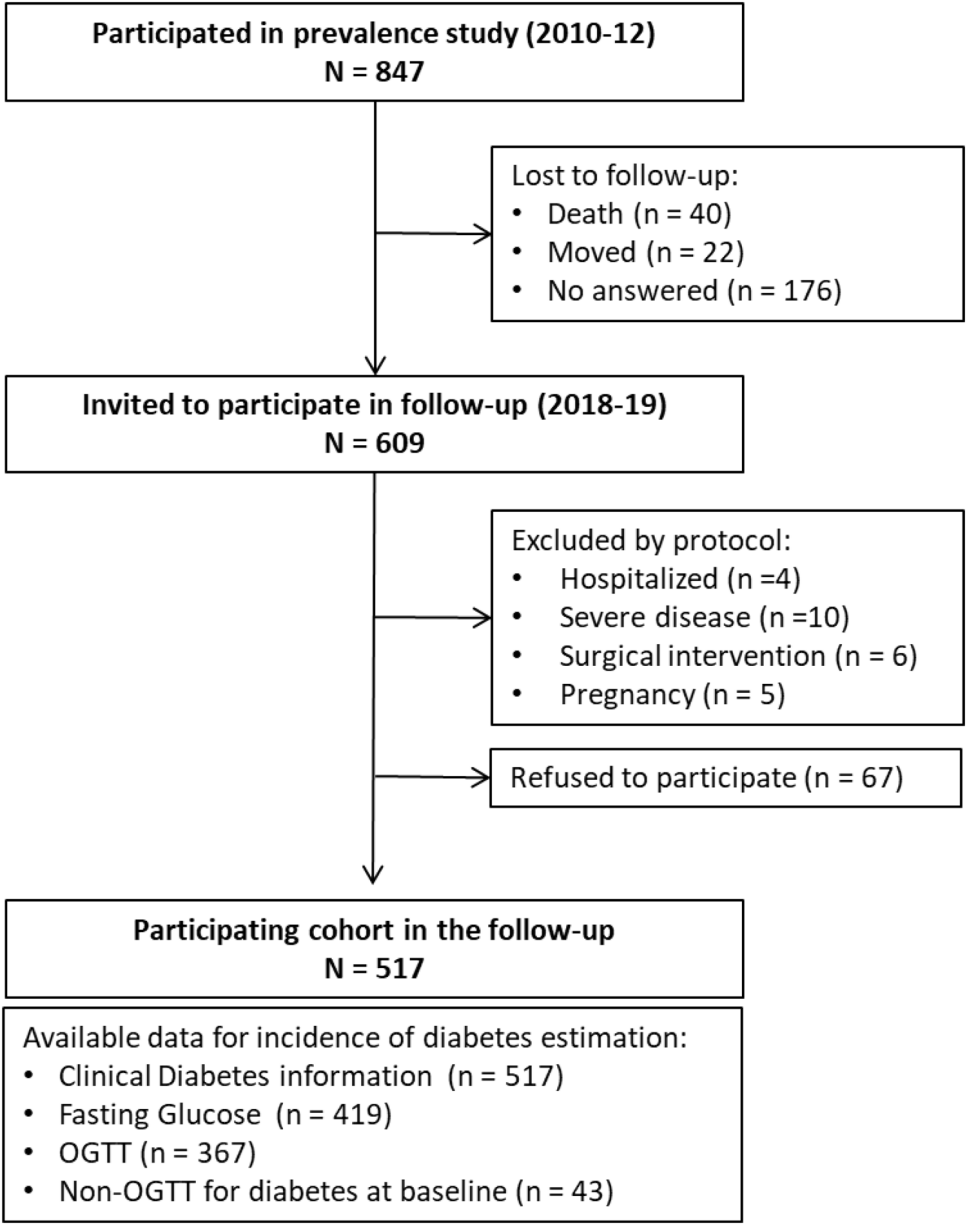
Participation flow chart.

A cross-sectional, cluster sampling design study was carried out during 2010–2012 to estimate the prevalence of diabetes in the Basque Country^10^. The Basque Country is a region of 7234 km^2^ in northern Spain with a population of 2,178,048 inhabitants, mostly Caucasian. Adults (≥ 18 years) were randomly selected from 20 public health centres representative of the Basque Country. A total of 847 people completed the baseline study. This cohort was reassessed in 2018–2019 after 7.1 years of follow-up (IQR 6.5–7.6). All individuals who took part in the baseline study (2010–2012) were invited to attend another clinical examination by letter and phone call to assist to medical centre. Among the 847 initial cases, 238 (loss to follow-up rate 28%) were not available (did not answer the phone, moved to another residence outside the area of interest or died), another 25 were excluded due to study protocol (hospitalized, severe disease, surgical intervention and pregnancy or recently delivered women) and 67 refused to participate. The resulting cohort consisted of 517 individuals (participation rate 85%) of whom 43 had diabetes at baseline, 367 (71%) underwent a 75 g OGTT, 52 (10%) accepted a fasting blood analysis and 98 (19%) only completed a basic questionnaire (Fig. 1).

The research was carried out in accordance with the Declaration of Helsinki (2008) of the World Medical Association. The study was approved by our local ethic committee, CEIC-E (Comité Etico de Investigación Clínica de Euskadi) and informed consent was obtained from all the participants.

### Procedures

The same methodology was used for both the prevalence and the reassessment study. In summary, all participants were invited to attend a medical examination at their health centre with a nurse specially trained for this project. Participants were required to answer a questionnaire on personal and family medical history and lifestyle. Physical examination included blood pressure (Hem-703 CP, Omron, Barcelona, Spain), weight, height, waist and hip circumferences measurements and, finally, a 75 g OGTT was also performed. The OGTT was not completed in people with capillary blood glucose measurements > 9.9 mmol/L (One Touch Select Plus, Lifescan, Johnson & Johnson, S.A., Madrid, Spain), or in individuals who had previous diabetes. When an OGTT was not possible, a fasting glucose analysis was carried out. People who did not want to fully participate in the study were requested to answer a basic questionnaire in order to collect information about pharmacological treatment (to determine the existence of clinical diabetes, hypertension or dyslipidaemia in treatment), the kind of diet they were following and self-reported weight.

All samples were centrifuged in situ 15 min after each blood extraction and transported daily for biochemical parameters analyses to the Clinical Biochemistry Laboratory of the Cruces University Hospital. Glucose, HDL-cholesterol and triglycerides were analysed in the ADVIA 2400 Chemistry analyser (Siemens Healthcare Diagnostics Inc, Deerfield, IL, USA), according to the corresponding reagent protocols. The LDL-cholesterol was calculated by Friedewald equation. Insulin was measured by chemiluminescence immunoassay in the LIAISON analyser (DiaSorin, Italy).

### Definition of cardiovascular risk factors

Diabetes and other categories of glucose metabolism disorders were classified according to the WHO criteria^11^. Diagnostic values for blood venous fasting glucose: NFG (Normal fasting glucose) < 6.1 mmol/L; IFG (Impaired fasting glucose) 6.1–7.0 mmol/L, Diabetes ≥ 7.0 mmol/L. Diagnostic values for blood venous glucose concentration at 120 min (75 g OGTT): NGT (Normal glucose tolerance) < 7.8 mmol/L; IGT (Impaired glucose tolerance) 7.8–11.1 mmol/L; Diabetes ≥ 11.1 mmol/L. People with isolated IFG or IGT and those with both disorders (IFG + IGT) were considered to have prediabetes. Insulin resistance was defined as homeostasis model assessment for insulin resistance (HOMA-IR) higher than three^26^.

The following variables, equally considered in baseline study, were also analysed: obesity defined as BMI ≥ 30 kg/m^2^; abdominal obesity defined as waist circumference (WC ≥ 94 cm in men and ≥ 80 cm in women) or waist-to-hip-ratio (WHR > 1 in men and > 0.85 in women); Hypertension defined as systolic pressure ≥ 140 mmHg and/or diastolic pressure ≥ 90 mmHg or patient under antihypertensive treatment; High LDL-cholesterol defined as values ≥ 3.9 mmol/L or patient under lipid-lowering treatment; High Triglycerides level defined as values ≥ 1.7 mmol/L; Low HDL-cholesterol defined as values < 1.03 mmol/L in men and < 1.29 mmol/L in women^27^.

### Statistical analysis

Qualitative variables were described as frequencies and percentages, and non-parametric quantitative variables as median and interquartile range (IQR: P_25_–P_75_). For comparisons, Chi-square test and Mann–Whitney U-test were applied as appropriate in each case. Participants with missing data in the variable of interest were excluded for statistical analysis.

A univariate binomial logistic regression model was performed to evaluate the association between several variables and diabetes. Variables with clinical relevance and *p*-values < 0.2 were then included in a multivariate binomial logistic regression model to assess the contribution of different variables in diabetes prediction. Two models were tested. The starting model A included all variables with *p* < 0.2 in the univariate analysis. In the model B, the same variables were included, except for the pre-diabetes status at baseline and the HOMA IR index. The two models were built manually. At each step of the modelling process, we repeatedly eliminated the variable with the highest *p-*value and we tested the model again until all remaining variables were significant (*p* < 0.05). Age and sex were kept in the final model regardless of their statistical significance because they are recognized predictors of the development of diabetes. Results were expressed as odds ratio (OR) and 95% confident interval (95% CI). Firth's method was applied in order to reduce the bias of maximum likelihood estimates due to rare events.

To compare the discrimination power of the two models (ability of the model to separate individuals who will develop diabetes from those who will not) the area under the receiver operating characteristic (AUC–ROC) curves and their 95% confidence intervals (95%CI) were calculated using standard techniques. The model with higher AUC–ROC curve is considered to have better discrimination. The Hosmer–Lemeshow test was assessed to describe the degree of adjustment between the predictions estimated by the model and the observed results (goodness-of-fit). The test indicates a good fit if the result is not significant, indicating that there are no statistically significant differences between the observed and expected values.

The incidence rates of diabetes in the cohort and according to the studied variables were calculated as numbers of events/person-time at risk for diabetes (people at risk were those without diabetes in the previous prevalence study). A constant incidence over time was assumed and was expressed per 1000 person-years. To enable comparisons with the national study, the estimation of the population incidence was adjusted for sex and age by direct method using the Spanish population as reference.

Statistical analyses were performed using R software version 4.0.1 (R Foundation for Statistical Computing). Results were considered statistically significant when *p* < 0.05.

## Abbreviations

IFG: Impaired fasting glucose
IGT: Impaired glucose tolerance
NFG: Normal fasting glucose
NGT: Normal glucose tolerance
WC: Waist circumference
WHR: Waist-to-hip ratio
